# Risk of transmission of foot-and-mouth disease by wild animals: infection dynamics in Japanese wild boar following direct inoculation or contact exposure

**DOI:** 10.1186/s13567-022-01106-0

**Published:** 2022-10-22

**Authors:** Katsuhiko Fukai, Rie Kawaguchi, Tatsuya Nishi, Mitsutaka Ikezawa, Manabu Yamada, Kingkarn Boonsuya Seeyo, Kazuki Morioka

**Affiliations:** 1 Exotic Disease Research Station, National Institute of Animal Health, National Agriculture and Food Research Organization, 6-20-1 Josui-honcho, Kodaira, Tokyo 187-0022 Japan; 2 National Institute of Animal Health, National Agriculture and Food Research Organization, 3-1-5 Kannondai, Tsukuba, Ibaraki 305-0856 Japan; 3 Regional Reference Laboratory for Foot and Mouth Disease in South East Asia, 1213/1, Moo11, Pakchong, 30130 Nakhornratchasima Thailand

**Keywords:** Experimental infection, foot-and-mouth disease, infection dynamics, pig, wild boar

## Abstract

Understanding of disease dynamics and viral shedding in wild boar and of the potential for disease spreading within wild boar and domestic pig populations is critical for developing effective control and eradication measures for foot-and-mouth disease (FMD). Accordingly, we infected experimentally wild boar and domestic pigs with FMD virus (FMDV) strains O/TAI/315/2016 and A/MOG/2013, and studied their susceptibility and viral transmissibility in both populations. Similar to FMDV-infected pigs, wild boar inoculated with both viruses exhibited vesicular lesions on their feet, snout, tongue and lip, although they did not show lameness. Further, inoculated wild boar were equally capable of transmitting the virus to all of their contact animals. While all contact pigs developed vesicular lesions after contact with inoculated animals, in contrast, no wild boar when exposed to the same infected animals showed obvious clinical signs. These results will be useful for further understanding of the critical roles in occurring and sustaining an FMD outbreak, and will be useful for establishing epidemiological surveillance programs and effective countermeasures for wild boar.

**Supplementary Information:**

The online version contains supplementary material available at 10.1186/s13567-022-01106-0.

## Introduction

Foot-and-mouth disease (FMD) is among the most contagious diseases, affecting cloven-hoofed domestic and wild animals, including cattle, water buffalo, sheep, goats and pigs [1]. FMD is caused by the FMD virus (FMDV), a member of the genus *Aphthovirus* within the family *Picornaviridae* [1]. FMDV serotypes are A, O, C, Asia1, SAT1, SAT2 and SAT3 [1], and topotypes, lineages and sublineages define the phylogenetic clustering of VP1 structural protein sequence of each serotype [2]. Clinically, FMD is characterized by vesicular lesions in the mouth, snout, feet and teats [3–5]. Manifestation of FMD and susceptibility to the disease vary depending on the animal species and virulence of the virus strain [3–5]. Transmission occurs via contact with infected animals, their secretions and excretions, animal products, aerosolized droplets, and mechanical vectors [3, 4]. FMDV multiplies quickly in infected animals although at variable degree among species, with one pig estimated to produce up to approximately 60-fold more airborne virus per day than sheep or cattle [3].

Numerous species of wild animals are susceptible to FMDV [5–10]. Because control measures such as vaccination and movement restriction cannot be applied to wild animals, in the case of an outbreak, introducing FMD to susceptible wild animals would further complicate eradication and control measures. Additionally, when the habitats of wild animals are near farms containing domestic animals, the former could become an infectious source of FMDV to the latter. In fact, FMD cases suspected of involving wild animals have been reported [11–18]. Furthermore, the presence of FMD in wild animals will affect the international trade of domestic animals and animal products. Detailed information on the wild animals, such as population size and geographical distribution, will need to be provided and measures put in place to prevent contact with domestic animals. Further, surveillance of wild animals will be required by trading partner countries, which could have significant economic impact [19, 20]. Therefore, control measures in wild animals are essential for both the eradication and control of FMD and international export strategies in the livestock sector.

The wild boar (*Sus scrofa*) is one of the most widely distributed wild mammals in the world. These animals inhabit 5 continents, but are concentrated in all regions of Eurasia [21]. Wild boar is classified into 16 subspecies based on the morphological characteristics of their skulls and facial bones. In addition, although coat color and body mass vary across subspecies, their ecology is similar [21]. Importantly, the habitats of wild boar overlap with regions prevalent in FMD, such as North Africa, the Middle East and Asia. In addition, domestic pigs (*Sus scrofa domesticus*), which belong to the same species as wild boar, produce larger amounts of aerosolized viruses than cattle and small ruminants, making them an important source of outbreaks [3, 22, 23]. Therefore, it is possible that wild boar could also be a source of FMD infection. However, studies of FMD and data on the transmissibility and clinical manifestations of the disease in wild boar are limited [24, 25].

To manage potential outbreaks of FMD in wild animals efficiently, it is important to identify infected animals as early as possible through appropriate surveillance programs and adopt established response strategies to control outbreaks. Therefore, understanding the disease dynamics and viral shedding in wild boar and the potential for disease transmission between wild boar and domestic pigs are critical for developing measures to control and eradicate FMD. Hence, in this study, we compared the transmission dynamics and transmissibility of FMDV in Japanese wild boar with those in domestic pigs.

## Materials and methods

### Facility

All experimental infections were performed in cubicles of approximately 14 m^2^ in a high-containment facility at the National Institute of Animal Health (NIAH). The cubicles were kept at 25 °C and provided 10 to 15 air changes per hour during the study period. The high-containment facility is compliant with a containment level for group 4 pathogens described in the OIE Manual of Diagnostic Tests and Vaccines for Terrestrial Animals 2021 [26].

### Virus

We used FMDV isolates of O/TAI/315/2016, belonging to serotype O, topotype ME-SA and linage Ind-2001e, and A/MOG/2013, belonging to serotype A, topotype ASIA and linage Sea-97. O/TAI/315/2016 isolate was obtained from a tongue epithelial sample from cattle in Songkhla province in Thailand in November 2016. The virus was initially isolated from primary lamb kidney cells and subsequently passaged twice in ZZ-R 127 and IB-RS-2 cells [27, 28]. Furthermore, the virus was additionally passaged once in ZZ-R 127 cells before use for experimental infections. A/MOG/2013 isolate was kindly supplied by the Pirbright Institute, UK, and passaged once in bovine thyroid cells, twice in BHK-21 cells [29] and once in IB-RS-2 cells before experimental infections.

### Experimental infections

We performed three experimental infections, as detailed below (Table 1). During the experimental infections, animals were given a commercial formulated feed for domestic pigs twice a day. Water was supplied using a water bucket and a water nipple. Pigs were able to drink water freely all day. Animals were sedated with 2 mg/kg of xylazine (Selactar, Bayer, Tokyo, Japan) and 10 mg/kg of ketamine (Ketalar, Sankyo, Tokyo, Japan) before virus inoculation. Intraoral inoculation was performed as previously described [30]. Briefly, sedated animals were placed on their backs on the floor, and their heads were held so that their muzzles faced the ceiling. Next, their mouths were opened, and the inoculum was deposited onto their tonsils using a 2.5-mL plastic syringe attached to gum tubing (inner diameter, 2 mm; length, 10 cm). Animals were kept in that position for a few minutes to prevent premature loss of the inoculum. Inoculated and contact animals were allowed to mix freely within the cubicle in which they were housed.

**Table 1:** **Experimental design**.

Experiment	Group	Treatment	Strain	Animal#
1	A	Inoculated domestic pig (intraorally)	O/TAI/315/2016	197, 198, 199
	B	Inoculated wild boar (intraorally)		1910, 1911
2	C	Inoculated wild boar (intraorally)	O/TAI/315/2016	191
		Contact wild boar		192
		Contact domestic pig		193
	D	Inoculated domestic pig (intraorally)		194
		Contact domestic pig		195
		Contact wild boar		196
3	E	Inoculated wild boar (intradermally)	A/MOG/2013	201
		Contact domestic pig		202, 203
	F	Inoculated domestic pig (intradermally)		204
		Contact wild boar		205, 206

Each group was examined in different cubicles.

In Experiment 1, three 2-month-old pigs (Pigs#197, 198 and 199, approximately 15 kg) and two Japanese wild boar (*Sus scrofa leucomystax*) captured in the field (Boar#1910 and 1911, age unknown, approximately 10 kg) were intraorally inoculated with 10^7.0^ 50% tissue culture infectious dose (TCID_50_) of O/TAI/315/2016. The pigs were housed in a cubicle separate from the wild boar (Groups A and B, Table 1). The animals were observed for 8 days after virus inoculation.

In Experiment 2, one 2-month-old wild boar raised in a farm (Boar#191, approximately 10 kg) and one 2-month-old pig (Pig#194, approximately 15 kg) were intraorally inoculated with 10^8.7^ TCID_50_ of O/TAI/315/2016 and housed separately in different cubicles. From day 1 post-inoculation (dpi), Boar#191 was housed with one 2-month-old wild boar raised in a farm (Boar#192, approximately 10 kg) and one 2-month-old pig (Pig#193, approximately 15 kg). Meanwhile, Pig#194 was housed with one 2-month-old pig (Pig#195, approximately 15 kg) and one 2-month-old wild boar raised in a farm (Boar#196, approximately 10 kg) at 1 dpi. Thus, each inoculated animal was housed with two contact animals in each cubicle (Groups C and D, Table 1).

In Experiment 3, one 2-month-old wild boar raised in a farm (Boar#201, approximately 15 kg) and one 2-month-old pig (Pig#204, approximately 15 kg) were intradermally inoculated with 10^7.0^ TCID_50_ of A/MOG/2013 and housed separately in different cubicles. From 0 dpi, Boar#201 was housed with two 2-month-old pigs (Pigs#202 and 203, approximately 15 kg), while Pig#204 was housed with two 2-month-old wild boar raised in a farm (Boar#205 and 206, approximately 10 kg). Thus, each inoculated animal was housed with two contact animals in each cubicle (Groups E and F, Table 1).

Clinical signs were scored as follows: lesions on each foot, 1 point; lesions in or around the mouth, 1 point; lesions in or around the snout, 1 point. Consequently, the maximum score per animal was 6. Once a lesion appeared at a site, the site was scored “positive” on all subsequent days, even if the lesion at that site eventually healed.

### Collection and preparation of clinical samples

Blood was collected from animals cervical veins into a vacuum blood collection tube (Venoject II, Terumo Corporation, Tokyo, Japan) and centrifuged to obtain sera. Oral swab samples were collected from oral cavities using a roll-shaped synthetic oral swab collector (Salivette, Sarstedt KK, Tokyo, Japan) and forceps. The oral swab samples were centrifuged and sterilized using a centrifugal filter unit (Ultrafree-MC, Merck Millipore, Darmstadt, Germany). Nasal swab samples were collected from nasal cavities using a cotton swab (Men-tip, JCB Industry Limited, Tokyo, Japan). The swabs were immersed in 10-time volumes (w/v) of Dulbecco’s Modified Eagle Medium/Nutrient Mixture F12 (DMEM/F12, Thermo Fisher Scientific, Waltham, MA, USA) and centrifuged, and the resulting supernatant was sterilized using a centrifugal filter unit (Ultrafree-CL, Merck Millipore). All clinical samples were collected daily from each animal during the experimental period except for blood in Experiment 2, which was collected at 1 to 3 days intervals.

### Cell culture, virus isolation and titration

LFBK-α_v_β_6_ cells were used for virus isolation and titration [31, 32]. The cells were maintained in DMEM/F12 supplemented with 10% fetal bovine serum.

For virus isolation and titration, first, ten-fold serial dilutions of the original clinical samples were prepared in tubes to determine virus titers. Second, 100 μL of each dilution was transferred to 4 wells of 96-well plates, followed by 100 μL of the LFBK-α_v_β_6_ cell suspension. Finally, the plates were incubated for 72 h at 37 °C in 5% CO_2_. Virus isolation and titration were performed on the same day as sample collection to minimize any decrease in virus titer due to cold storage or freezing and thawing processes. The cells were examined under a light microscope for any cytopathic effects (CPE). The specificity of a CPE was confirmed using a monoclonal antibody-based sandwich directed enzyme-linked immunosorbent assay or reverse transcription-polymerase chain reaction (RT-PCR), as described previously [33, 34]. Virus titers were calculated using the Reed-Muench method [35]. All clinical samples were subjected to the virus isolation and titration procedures.

### RNA extraction and RT-PCR

Viral RNAs were extracted from clinical samples using the High Pure Viral RNA Kit (Roche Diagnostics, Basel, Switzerland) according to the manufacturer’s instructions. FMDV-specific genes were detected from extracted RNA samples using the RT-PCR method described previously [34]. All clinical samples were subjected to the RNA extraction and RT-PCR procedures.

### Antibody detection from sera

The virus neutralization test (VNT) was performed using LFBK-α_v_β_6_ cells as described previously [36]. O/TAI/315/2016 and A/MOG/2013 were used as antigens in the VNT to determine the animals’ antibody response to each isolate. Antibody responses to structural proteins of FMDV were detected using the PrioCHECK FMDV Type O Antibody ELISA Kit (Applied Biosystems, Waltham, MA, USA) and PrioCHECK FMDV Type A Antibody ELISA Kit (Applied Biosystems).

### Tissue samples

During necropsy of the inoculated and contact animals in Experiment 1 and 2, tissue samples were taken from the tongue, soft palate tonsil, soft palate, oropharynx, nasopharynx, larynx, trachea, esophagus, mandibular gland, parotid gland, intraoral salivary gland, mandibular lymph node (LN), parotid LN, lateral retropharyngeal LN, superficial cervical LN, inguinal LN, thymus, liver, spleen, kidney, heart, lung, stomach, small intestine, large intestine, lower lip, and skin of the snout, coronary band, and heel bulb of each animal. For histopathology, the tissues were fixed in 10% neutral phosphate-buffered formalin, processed according to routine procedures, and embedded in paraffin wax. Sections were stained with hematoxylin and eosin. For immunohistochemistry, dewaxed sections were processed using the universal immuno-enzyme polymer method with the HISTONE simple stain Max PO (M) kit (Nichirei, Tokyo, Japan) according to the manufacturer’s instructions. Sections were labeled using a monoclonal antibody specific for FMDV (16D6, diluted 1:8, NIAH, Japan) and counterstained with hematoxylin.

## Results

### Experiment 1

**Figure 1:**
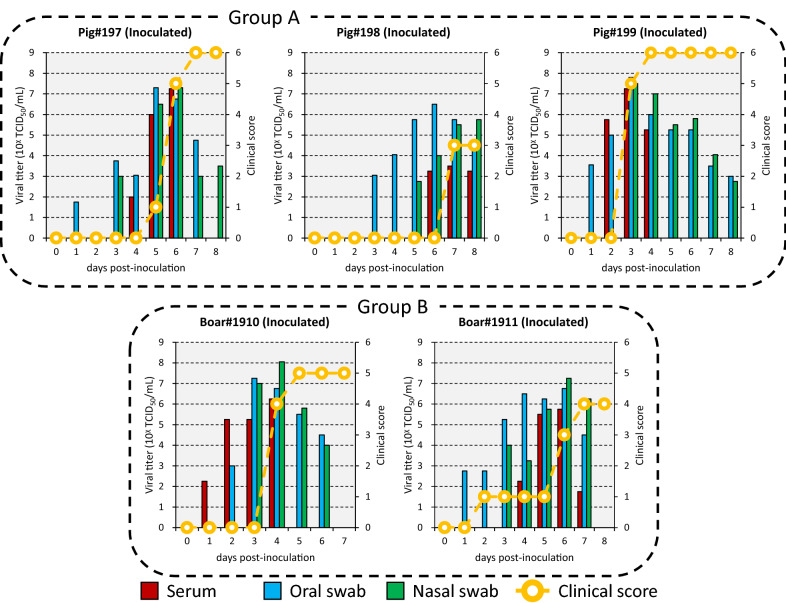
**Viremia, virus excretion and clinical score in inoculated animals intraorally inoculated with O/TAI/315/2016.** Groups A and B were housed in different cubicles.

Pigs#197–199 showed vesicular lesions on the feet and lips from 3 to 7 dpi, and had a total clinical score of 3 to 6 (Figure 1). Viremia ranging from 10^2.0^ to 10^7.3^ TCID_50_/mL and virus excretion ranging from 10^1.8^ to 10^7.8^ TCID_50_/mL were noted at 2 to 8 and 1 to 8 dpi, respectively (Figure 1). Viremia and virus excretion were confirmed by RT-PCR (Additional file 1). Antibodies were detected in Pigs#197 and 199 from 7 to 8 dpi by VNT and ELISA but not in Pig#198 (Additional file 1).

**Figure 2:**
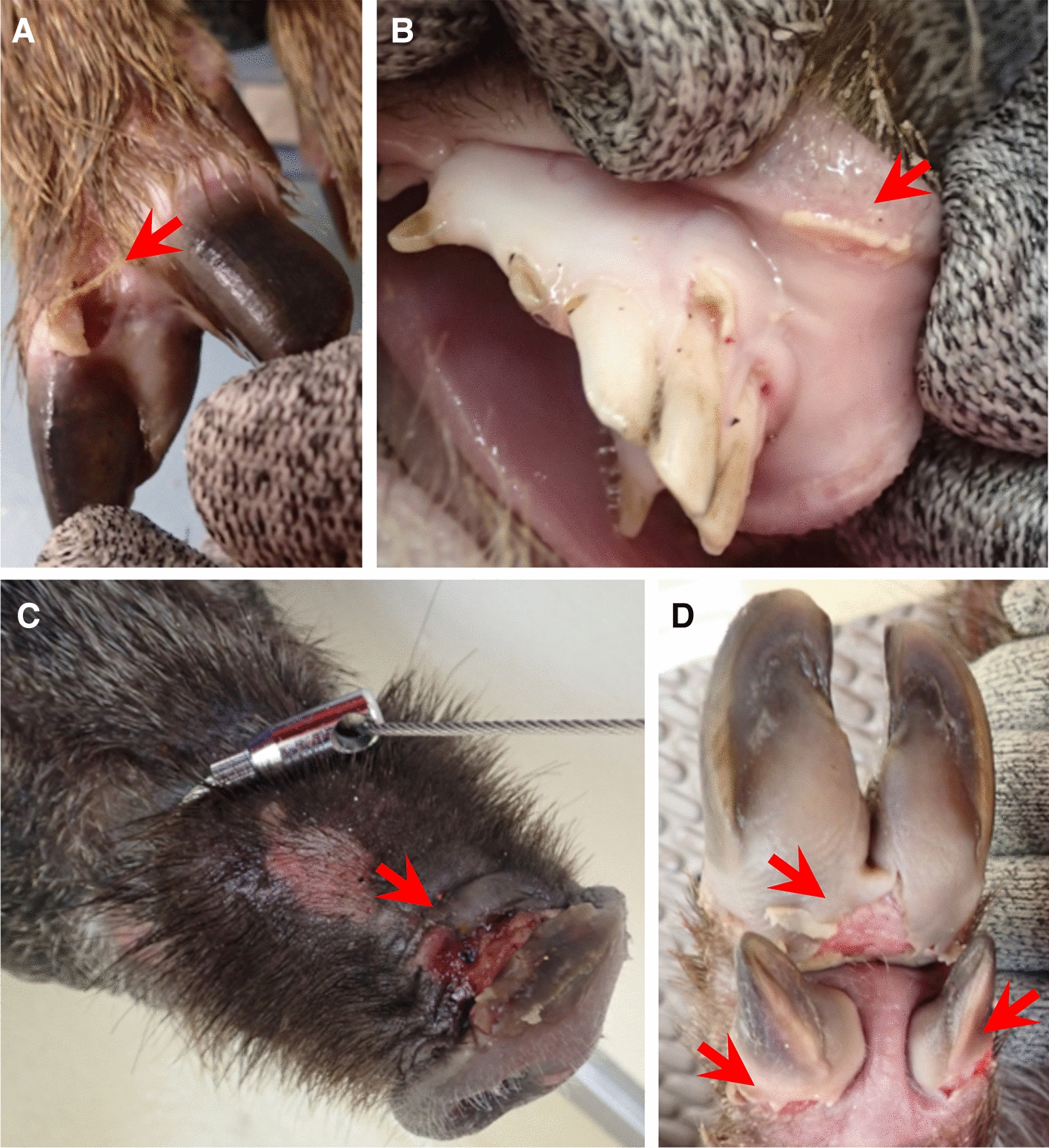
**Vesicular lesions in wild boar**. **A** Boar#1910, right rear foot, 6 dpi; **B** Boar#1910, lip, 7 dpi; **C** Boar#201, snout, 5 dpi; **D** Boar#201, left front foot, 5 dpi. Arrows indicate vesicular lesions.

**Figure 3:**
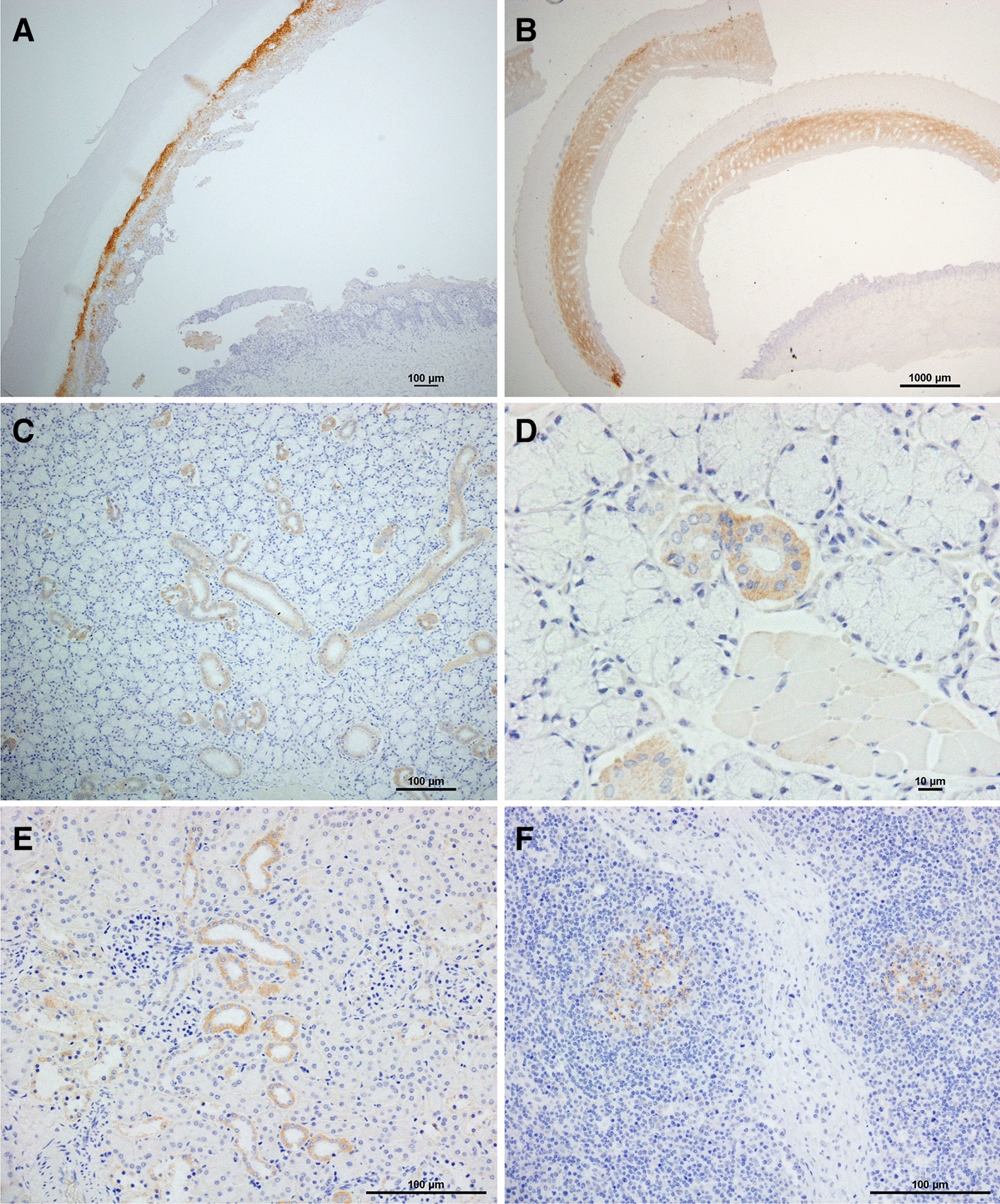
**Immunohistochemical detection of FMDV antigens at 7 dpi in tissues from Boar#1910 intraorally inoculated with O/TAI/315/2016.** **A** skin of coronary band. FMDV antigens are detected in the stratum spinosum of the vesicular epidermis. **B** skin of heel bulb. FMDV antigens are detected in the stratum spinosum of the vesicular epidermis. **C** mandibular gland. FMDV antigens are detected in ductal epithelium cells. D salivary gland. FMDV antigens are detected in ductal epithelium cells. **E** kidney. FMDV antigens are detected in the epithelium cells of the collecting ducts. **F** inguinal lymph node. FMDV antigens are detected in the lymphoid follicles.

Boar#1910 and 1911 showed vesicular lesions on the feet, snout, tongue and lip from 2 to 7 dpi (Figures 1 and 2). Histologically, vesicular lesions and antigens were also observed in the lip and skin of the snout, coronary band and heel bulb (Figure 2 and Additional file 2). In addition, viral antigens were also detected in the mandibular, parotid and intraoral salivary glands, superficial and inguinal LNs, and kidney (Figure 3). The boar had a total clinical score of 4 to 5 (Figure 1). Viremia ranging from 10^1.8^ to 10^6.3^ TCID_50_/mL and virus excretion ranging from 10^2.8^ to 10^8.1^ TCID_50_/mL were noted at 1 to 7 and 1 to 7 dpi, respectively (Figure 1). Viremia and virus excretion were confirmed by RT-PCR (Additional file 1). Antibodies were detected in Boar#1910 at 7 dpi by VNT and ELISA but not in Boar#1911 (Additional file 1). Boar#1910 died at 7 dpi due to an accident during blood collection.

### Experiment 2

**Figure 4:**
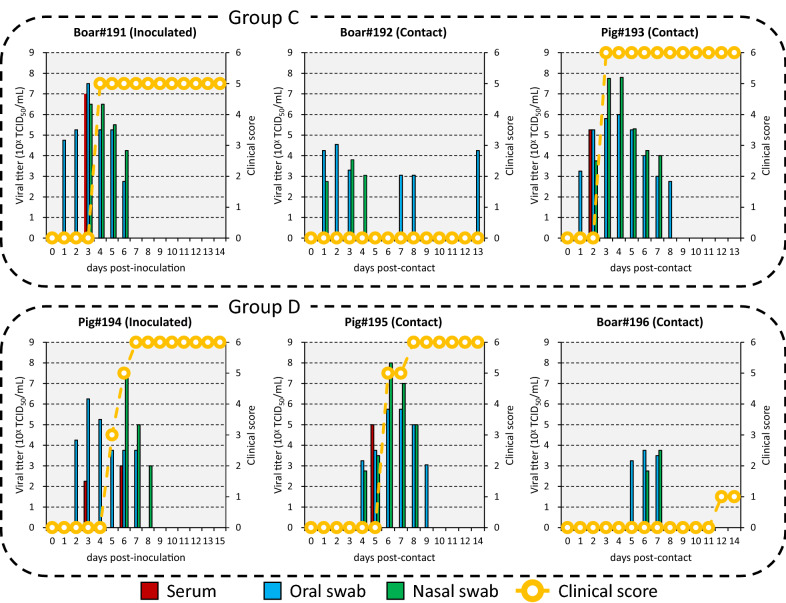
**Viremia, virus excretion and clinical score in animals intraorally inoculated with O/TAI/315/2016 and in contact animals.** Groups C and D were housed in different cubicles.

Inoculated Boar#191 and Pig#194 showed vesicular lesions on the feet and lip from 4 to 7 dpi. Histologically, vesicular lesions and antigens were also observed in the tongue and the skin of the coronary band and heel bulb (Additional file 3). The animals had a total clinical score of 5 to 6 (Figure 4). Viremia ranging from 10^2.3^ to 10^7.0^ TCID_50_/mL and virus excretion ranging from 10^2.8^ to 10^7.5^ TCID_50_/mL were noted at 3 to 6 and 1 to 8 dpi, respectively (Figure 4). Viremia and virus excretion were confirmed by RT-PCR (Additional file 4). Antibodies were detected from 6 to 9 dpi by VNT and ELISA (Additional file 4).

Although Pig#193, which was in contact with inoculated Boar#191, showed vesicular lesions on the feet, snout, tongue and lip from 3 days post-contact (dpc) and had a total clinical score of 6, Boar#192, which was also in contact with inoculated Boar#191, showed no vesicular lesions during the experimental period (Figure 4). Histologically, vesicular lesions were also observed in the skin of the coronary band and heel bulb of Pig#193 but not that of Boar#192 (Additional file 3). Viremia of 10^5.3^ TCID_50_/mL was observed at 2 dpc in Pig#193, while no viremia was noted in Boar#192 (Figure 4). Meanwhile, virus excretion ranging from 10^2.8^ to 10^7.8^ TCID_50_/mL was observed at 1 to 13 dpc in both animals (Figure 4). Findings of viremia in only Pig#193 and virus excretion in both animals were confirmed by RT-PCR (Additional file 4). Antibodies were detected from 8 to 13 dpc in both animals by VNT and ELISA (Additional file 4).

Similarly, although Pig#195, which was in contact with inoculated Pig#194, showed vesicular lesions on the feet and lip from 6 to 8 dpc and had a total clinical score of 6, Boar#196, which was also in contacted with inoculated Pig#194, showed a weak lesion on the foot at 12 dpc and had a total clinical score of just 1 (Figure 4). Histologically, vesicular lesions and antigens were also observed in the skin of the snout, coronary band and heel bulb of Pig#195 but not that of Boar#196 (Additional file 3). Viremia of 10^5.0^ TCID_50_/mL was observed at 5 dpc in Pig#195, while, no viremia was noted in Boar#196 (Figure 4). Virus excretion ranging from 10^2.8^ to 10^8.0^ TCID_50_/mL was observed at 4 to 9 dpc in both animals (Figure 4). Findings of viremia in Pig#195 and virus excretion in both animals were confirmed by RT-PCR (Additional file 4). Antibodies were detected from 11 dpc in Pig#195 by VNT and ELISA but not in Boar#196 (Additional file 4).

### Experiment 3

**Figure 5:**
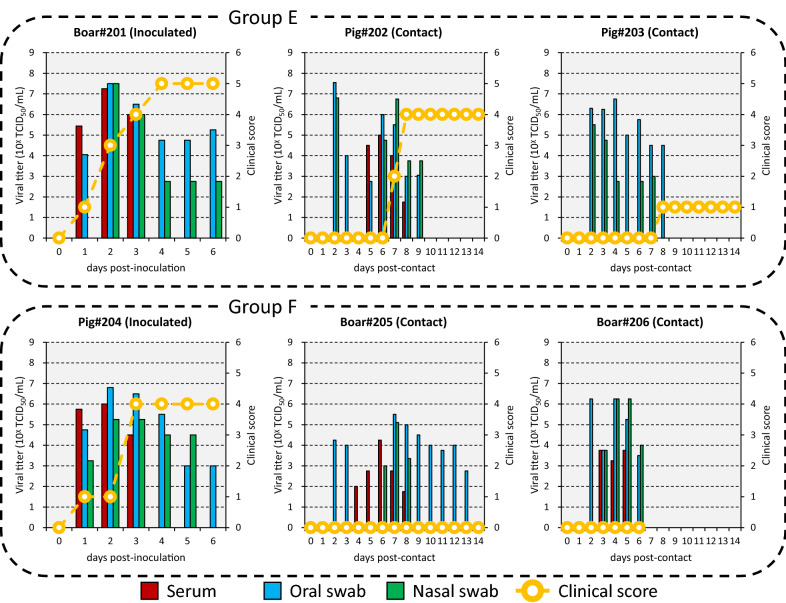
**Viremia, virus excretion and clinical score in inoculated animals intradermally inoculated with A/MOG/2013 and in contact animals.** Groups E and F were housed in different cubicles.

Inoculated Boar#201 and Pig#204 showed vesicular lesions on the feet and snout from 1 to 4 dpi (Figures 2 and 5). The animals had a total clinical score of 4 to 5 (Figure 5). Viremia ranging from 10^4.5^ to 10^7.3^ TCID_50_/mL and virus excretion ranging from 10^2.8^ to 10^7.5^ TCID_50_/mL were noted at 1 to 3 and 1 to 6 dpi, respectively (Figure 5). Viremia and virus excretion were confirmed by RT-PCR (Additional file 5). While antibodies were detected from 4 to 6 dpi in both animals by VNT, they could not be detected in Pig#204 by ELISA (Additional file 5). The inoculated animals were euthanized for ethical reasons at 6 dpi.

Although Pig#202, which was in contact with inoculated Boar#201, showed vesicular lesions on the feet from 7 dpc and had a total clinical score of 4, Pig#203, which was also in contact with inoculated Boar#201, showed a weak lesion on only the left front foot from 8 dpc and had a total clinical score of just 1 (Figure 5). Viremia ranging from 10^1.8^ to 10^5.0^ TCID_50_/mL was observed from 5 to 8 dpc in Pig#202 but not in Pig#203 (Figure 5). Virus excretion ranging from 10^2.8^ to 10^7.6^ TCID_50_/mL was noted at 2 to 9 dpc in both animals (Figure 5). Findings of viremia in Pig#202 and virus excretion in both animals were confirmed by RT-PCR (Additional file 5). Further, while antibodies were detected from 9 to 11 dpc in both animals by VNT, they could not be detected in Pig#203 by ELISA (Additional file 5).

Neither Boar#205 nor 206, which were in contact with inoculated Pig#204, showed any vesicular lesions during the experimental period (Figure 5). Viremia ranging from 10^1.8^ to 10^4.3^ TCID_50_/mL and virus excretion ranging from 10^2.8^ to 10^6.3^ TCID_50_/mL were noted from 2 to 13 dpc (Figure 5). Viremia and virus excretion were confirmed in both animals by RT-PCR (Additional file 5). Antibodies were detected from 8 to 10 dpc in Boar#205 by VNT and ELISA but not in Boar#206 (Additional file 5). Boar#206 died due to an unknown reason at 6 dpc.

## Discussion

Our comparison of the transmission dynamics and transmissibility of FMDV in Japanese wild boar and domestic pigs showed that: (1) wild boar in Japan were susceptible to FMDV by intraoral and intradermal inoculation; (2) infected wild boar and domestic pigs shed similar quantities of virus and were equally capable of transmitting the virus to their contact animals; (3) compared to domestic pigs, wild boar exhibited delayed or weak clinical signs, including difficult-to-detect lesions and a lack of lameness.

We showed that wild boar in Japan were susceptible to FMDV strains O/TAI/315/2016 and A/MOG/2013 by intraoral and intradermal inoculation. Although lameness was evident in domestic pigs when they attempted to put weight on the affected feet, such clinical signs were absent from inoculated wild boar. Instead, clinical signs in inoculated wild boar were vesicular lesions on the feet, snout, tongue and lip. While we were able to identify these lesions under controlled conditions in the present study, in the wild, lesions on the feet would be difficult to detect because the wild boar had long dark hair and black skin. In a previous study, clinical signs in feral pigs took longer to appear and were more difficult to detect compared to those in domestic pigs [25]. In addition, infected wild boar displayed severe foot lesions, which did not appear to impair their mobility [24]. These data indicate that clinical signs of FMD could be difficult to detect in wild boar in the field. They also suggest that the infected animals could remain mobile and spread FMDV, which is problematic for controlling an outbreak of FMD.

While all contact pigs developed vesicular lesions during the experimental period, none of the contact wild boar exposed to either an inoculated pig or wild boar did so. The reasons for the lack of apparent vesicular lesions in contact wild boar compared to domestic pigs is unclear. Further, this finding is inconsistent with previous studies, where, although the onset of clinical signs in domestic pigs via direct contact with inoculated animals was quicker than that in feral pigs and wild boar, all contact animals showed apparent vesicular lesions [24, 25]. The discrepancies between the present study and previous studies may be due to the difference in subspecies of the animals and FMDV strains tested. Nevertheless, the difficulty in detecting clinical signs in wild boar has important implications for observational surveillance programs, which are typically based on clinical detection.

In this study, virus shedding in oral and nasal swab samples was first detected 1 to 3 dpi in inoculated animals. This onset of viral shedding was confirmed using RT-PCR, indicating that inoculated animals shed the virus through their oral and nasal passages soon after they were infected with FMDV and often before showing clinical signs. Similar results have been reported in previous studies [24, 25]. Another study showed that domestic pigs can be infected intraorally with a minimum of 10^3^ TCID_50_ of FMDV [30]. In the present study, both inoculated wild boar and contact wild boar that showed no clinical signs shed more than 10^3^ TCID_50_/mL of virus into oral and nasal swab samples. Specifically, inoculated wild boar and domestic pigs shed similar quantities of virus and were equally capable of transmitting the virus to contact animals in this study. These results suggest that, in addition to remaining mobile regardless of whether they develop vesicular lesions, FMDV-infected wild boar excrete sufficient amounts of virus to transmit it to contact animals. This is a problem for controlling the virus as it can lead to extensive spread of FMD in a region.

Although we did not perform viral titration in this study, we detected viral antigens in the vesicular lesions of wild boar and contact animals, suggesting that the virus was present in as many boars as pigs. This finding may provide insight into whether wild boar carcasses could be a source of FMDV infection, as while a few reports have examined the potential of FMDV-infected pig carcasses as a source of infection for susceptible animals, no studies have been conducted in wild boar. In one study in pigs [37], live FMDV was isolated from the muscles of pigs that were euthanized during the acute infection period and stored at 4 °C until 5 days after death. The same study also isolated live FMDV continuously from the vesicle epithelium of pigs euthanized during the same period until 77 days postmortem, and reported a virus titer of approximately 10^4^ TCID_50_/g [37]. The authors calculated that the half-life of the virus in the vesicular epithelium was 128 days, and that the time needed for it to be totally eliminated was 203 days [37]. However, an earlier report suggested that FMDV-infected pig carcasses could be a source of infection for a long period of time during the cold season in temperate climate zones. This was the case in the outbreak in South Korea in November 2010, when a cold wave prevented virus decontamination, leading to prolonged spread of the virus [38]. Therefore, if an outbreak occurs in wild boar and a wild boar dies in the acute infection phase for any reason, its carcass may be a prolonged source of infection, as is the case with pigs, which belong to the same species as wild boar.

In addition to the acute phase, a study that isolated live FMDV from several tissues collected from pigs that were euthanized at 17 dpi [39] suggests that pigs in the convalescent phase could also be a source of infection. Consistent with this, we detected viral antigens in several tissues in the second week after infection in this study. Although the period for which live FMDV in tissues has only been elucidated for bone marrows [37], these findings suggest that the tissues of FMDV-infected wild boar may be sources of infection; however, the degree likely depends on each tissue original viral titer.

In conclusion, we showed that while wild boar and domestic pigs in Japan were susceptible to FMDV by intraoral and intradermal inoculation, shed similar quantities of virus and were equally capable of transmitting the virus to contact animals, wild boar exhibited delayed or weak clinical signs. Increased vigilance is thus required when conducting FMD surveillance in wild boar. Control measures for FMD in wild boar could be more difficult to implement, making surveillance programs that emphasize early detection particularly important to minimize spread of FMD around infected farms in an outbreak.

## Supplementary Information

**Additional file 1**. **Detection of viral RNA in clinical samples by RT-PCR and of antibodies by VNT and ELISA in animals intraorally inoculated with O/TAI/315/2016 in Experiment 1**.

**Additional file 2.** **Antigen detection in tissue samples from wild boar intraorally inoculated with O/TAI/315/2016 in Experiment 1**.

**Additional file 3.** **Antigen detection in tissue samples from animals intraorally inoculated with O/TAI/315/2016 and in contact animals in Experiment 2**.

**Additional file 4.** **Detection of viral RNA in clinical samples by RT-PCR and of antibodies by VNT and ELISA in animals intraorally inoculated with O/TAI/315/2016 and in contact animals in Experiment 2**.

**Additional file 5.** **Detection of viral RNA in clinical samples by RT-PCR and of antibodies by VNT and ELISA in animals intradermally inoculated with A/MOG/2013 and in contact animals in Experiment 3**.
