# Supplementary material for: Risk of transmission of foot-and-mouth disease by wild animals: infection dynamics in Japanese wild boar following direct inoculation or contact exposure
Source: Vet Res. 2022 Oct 22;53:86. doi: 10.1186/s13567-022-01106-0 (PMC9587633; doi:10.1186/s13567-022-01106-0)
Supplement: Supplementary file 1 — Additional file 1. Detection of viral RNA in clinical samples by RT-PCR and of antibodies by VNT and ELISA in animals intraorally inoculated with O/TAI/315/2016 in Experiment 1. [file 13567_2022_1106_MOESM1_ESM.docx]

**Additional file 1 Detection of viral RNA in clinical samples by RT-PCR and of antibodies by VNT and ELISA in animals intraorally inoculated with O/TAI/315/2016 in Experiment 1**

| Animal | Clinical sample and assay | Days post-inoculation | | | | | | | | |
| --- | --- | --- | --- | --- | --- | --- | --- | --- | --- | --- |
|  |  | 0 | 1 | 2 | 3 | 4 | 5 | 6 | 7 | 8 |
| Pig#197 | Serum | -/-^a^ | -/- | -/- | -/- | 2.00/+^b^ | 6.00/+ | 7.25/+ | -/+ | -/- |
|  | Oral swab | -/- | 1.75/- | -/- | 3.75/+ | 3.05/+ | 7.30/+ | 6.75/+ | 4.75/+ | -/+ |
|  | Nasal swab | -/- | -/- | -/- | 3.00/+ | -/+ | 6.50/+ | 7.30/+ | 3.00/+ | 3.50/+ |
|  | VNT^c^ | <4 | <4 | <4 | <4 | <4 | <4 | <4 | 4^d^ | 8 |
|  | ELISA | - | - | - | - | - | - | - | - | +^e^ |
|  | Clinical score | 0 | 0 | 0 | 0 | 0 | 1^f^ | 5 | 6 | 6 |
| Pig#198 | Serum | -/- | -/- | -/- | -.- | -/- | -/- | 3.25/+ | 3.50/+ | 3.25/+ |
|  | Oral swab | -/- | -/- | -/- | 3.05/+ | 4.05/+ | 5.75/+ | 6.50/+ | 5.75/+ | 4.50/+ |
|  | Nasal swab | -/- | -/- | -/- | -/+ | -/+ | 2.75/+ | 4.00/+ | 5.50/+ | 5.75/+ |
|  | VNT | <4 | <4 | <4 | <4 | <4 | <4 | <4 | <4 | <4 |
|  | ELISA | - | - | - | - | - | - | - | - | - |
|  | Clinical score | 0 | 0 | 0 | 0 | 0 | 0 | 0 | 3 | 3 |
| Pig#199 | Serum | -/- | -/- | 5.75/+ | 7.25/+ | 5.25/+ | -/+ | -/- | -/- | -/- |
|  | Oral swab | -/- | 3.55/+ | 5.00/+ | 7.80/+ | 6.00/+ | 5.25/+ | 5.25/+ | 3.50/+ | 3.00/+ |
|  | Nasal swab | -/- | -/- | -/- | 7.50/+ | 7.00/+ | 5.50/+ | 5.80/+ | 4.05/+ | 2.75/+ |
|  | VNT | <4 | <4 | <4 | <4 | <4 | <4 | <4 | <4 | 11 |
|  | ELISA | - | - | - | - | - | - | + | + | + |
|  | Clinical score | 0 | 0 | 0 | 5 | 6 | 6 | 6 | 6 | 6 |
| Boar#1910 | Serum | -/- | 2.25/+ | 5.25/+ | 5.25/+ | 6.25/+ | -/+ | -/- | -/- | NS^g^ |
|  | Oral swab | -/- | -/- | 3.00/+ | 7.25/+ | 6.75/+ | 5.50/+ | 4.50/+ | -/+ | NS |
|  | Nasal swab | -/- | -/- | -/- | 7.00/+ | 8.05/+ | 5.80/+ | 4.00/+ | -/+ | NS |
|  | VNT | <4 | <4 | <4 | <4 | <4 | <4 | <4 | 8 | NS |
|  | ELISA | - | - | - | - | - | - | - | + | NS |
|  | Clinical score | 0 | 0 | 0 | 0 | 4 | 5 | 5 | 5 | NS |
| Boar#1911 | Serum | -/- | -/- | -/- | -/- | 2.25/- | 5.50/+ | 5.75/+ | 1.75/+ | -/+ |
|  | Oral swab | -/- | 2.25/+ | 2.75/+ | 5.25/+ | 6.50/+ | 6.25/+ | 6.75/+ | 4.50/+ | -/+ |
|  | Nasal swab | -/- | -/- | -/- | 4.00/+ | 3.25/- | 5.75/+ | 7.25/+ | 6.25/+ | -/- |
|  | VNT | NS | NS | <4 | <4 | <4 | <4 | <4 | <4 | <4 |
|  | ELISA | - | - | - | - | - | - | - | - | - |
|  | Clinical score | 0 | 0 | 1 | 1 | 1 | 1 | 3 | 4 | 4 |

^a^ Results of virus isolation and RT-PCR assay were shown sequentially. Virus titers were shown as 10^χ^ TCID_50_/mL. "+" means positive results in the RT-PCR assay. "-" means negative results in virus isolation and RT-PCR assay.

^b^ Days on which virus isolation and/or RT-PCR were positive are colored orange.

^c^ Virus neutralization test.

^d^ Days on which VNT was positive are colored yellow.

^e^ Days on which ELISA was positive are colored green.

^f^ Days on which clinical signs were scored are colored blue.

^g^ Not sampled.
