# Supplementary material for: Risk of transmission of foot-and-mouth disease by wild animals: infection dynamics in Japanese wild boar following direct inoculation or contact exposure
Source: Vet Res. 2022 Oct 22;53:86. doi: 10.1186/s13567-022-01106-0 (PMC9587633; doi:10.1186/s13567-022-01106-0)
Supplement: Supplementary file 2 — Additional file 2. Antigen detection in tissue samples from wild boar intraorally inoculated with O/TAI/315/2016 in Experiment 1. [file 13567_2022_1106_MOESM2_ESM.docx]

**Additional file 2 Antigen detection in tissue samples from wild boar intraorally inoculated with O/TAI/315/2016 in Experiment 1**

| Tissue | Animal | |
| --- | --- | --- |
|  | Boar#1910 | Boar#1911 |
| Tongue | -/-^a^ | -/- |
| Soft palate tonsil | -/- | -/- |
| Soft palate | -/- | -/- |
| Oropharynx | -/- | -/- |
| Nasopharynx | -/- | -/- |
| Larynx | -/- | -/- |
| Trachea | -/- | -/- |
| Esophagus | -/- | -/- |
| Mandibular gland | -/+ | -/- |
| Parotid gland | -/+ | -/- |
| Intraoral salivary gland | -/+ | -/- |
| Mandibular LN^b^ | -/- | -/- |
| Parotid gland | -/- | -/- |
| Lateral retropharyngeal LN | -/- | -/- |
| Superficial cervical LN | -/+ | -/- |
| Inguinal LN | -/+ | -/- |
| Thymus | NS^c^ | NS |
| Liver | -/- | -/- |
| Spleen | -/- | -/- |
| Kidney | -/+ | -/+ |
| Heart | -/- | -/- |
| Lung | -/- | -/- |
| Stomach | -/- | -/- |
| Small intestine | -/- | -/- |
| Large intestine | -/- | -/- |
| Lower lip | +/- | +/- |
| Snout skin | +/+ | +/+ |
| Coronary band skin | +/+ | +/+ |
| Heel bulb skin | +/+ | +/+ |

^a^ +/+: positive for necrotic lesion on histology/immunohistochemistry. Samples positive for viral antigens using both methods are colored orange, and those positive using one method are colored yellow.

^b^ Lymph node.

^c^ Not sampled.
