# Supplementary material for: Risk of transmission of foot-and-mouth disease by wild animals: infection dynamics in Japanese wild boar following direct inoculation or contact exposure
Source: Vet Res. 2022 Oct 22;53:86. doi: 10.1186/s13567-022-01106-0 (PMC9587633; doi:10.1186/s13567-022-01106-0)
Supplement: Supplementary file 3 — Additional file 3. Antigen detection in tissue samples from animals intraorally inoculated with O/TAI/315/2016 and in contact animals in Experiment 2. [file 13567_2022_1106_MOESM3_ESM.docx]

**Additional file 3 Antigen detection in tissue samples from animals intraorally inoculated with O/TAI/315/2016 and in contact animals in Experiment 2**

| Tissue | Animal | | | | | |
| --- | --- | --- | --- | --- | --- | --- |
|  | Boar#191 | Boar#192 | Pig#193 | Pig#194 | Pig#195 | Boar#196 |
| Tongue | +/-^a^ | -/- | -/- | -/- | -/- | -/- |
| Soft palate tonsil | -/- | -/- | -/- | -/- | -/- | -/- |
| Soft palate | -/- | -/- | NS^b^ | NS | NS | -/- |
| Oropharynx | -/- | -/- | NS | NS | NS | -/- |
| Nasopharynx | -/- | -/- | NS | NS | NS | -/- |
| Larynx | -/- | -/- | NS | NS | NS | -/- |
| Trachea | -/- | -/- | NS | NS | NS | -/- |
| Esophagus | -/- | -/- | NS | NS | NS | -/- |
| Mandibular gland | -/- | -/- | NS | NS | NS | -/- |
| Parotid gland | -/- | -/- | NS | NS | NS | -/- |
| Intraoral salivary gland | -/- | -/- | NS | NS | NS | -/- |
| Mandibular LN^c^ | -/- | -/- | NS | NS | NS | -/- |
| Parotid gland | -/- | -/- | NS | NS | NS | -/- |
| Lateral retropharyngeal LN | -/- | -/- | NS | NS | NS | -/- |
| Superficial cervical LN | -/- | -/- | NS | NS | NS | -/- |
| Inguinal LN | -/- | -/- | NS | NS | NS | -/- |
| Thymus | -/- | -/- | NS | NS | NS | -/- |
| Liver | -/- | -/- | NS | NS | NS | -/- |
| Spleen | -/- | -/- | -/- | -/- | -/- | -/- |
| Kidney | -/- | -/- | NS | NS | NS | -/- |
| Heart | -/- | -/- | -/- | -/- | -/- | -/- |
| Lung | -/- | -/- | NS | NS | NS | -/- |
| Stomach | -/- | -/- | NS | NS | NS | -/- |
| Small intestine | -/- | -/- | NS | NS | NS | -/- |
| Large intestine | -/- | -/- | NS | NS | NS | -/- |
| Lower lip | -/- | -/- | -/- | -/- | -/- | -/- |
| Snout skin | -/- | -/- | -/- | -/- | +/- | -/- |
| Coronary band skin | +/+ | -/- | +/- | +/- | +/+ | -/- |
| Heel bulb skin | +/+ | -/- | +/- | +/- | +/+ | -/- |

^a^ +/+: positive for necrotic lesion on histology/immunohistochemistry. Samples positive for viral antigens using both methods are colored orange, and those positive using one method are colored yellow.

^b^ Not sampled.

^c^ Lymph node.
