# Supplementary material for: Risk of transmission of foot-and-mouth disease by wild animals: infection dynamics in Japanese wild boar following direct inoculation or contact exposure
Source: Vet Res. 2022 Oct 22;53:86. doi: 10.1186/s13567-022-01106-0 (PMC9587633; doi:10.1186/s13567-022-01106-0)
Supplement: Supplementary file 4 — Additional file 4. Detection of viral RNA in clinical samples by RT-PCR and of antibodies by VNT and ELISA in animals intraorally inoculated with O/TAI/315/2016 and in contact animals in Experiment 2. [file 13567_2022_1106_MOESM4_ESM.docx]

**Additional file 4 Detection of viral RNA in clinical samples by RT-PCR and of antibodies by VNT and ELISA in animals intraorally inoculated with O/TAI/315/2016 and in contact animals in Experiment 2**

| Animal | Clinical sample and assay | Days post-inoculation or days post-contact | | | | | | | | | | | | | | |
| --- | --- | --- | --- | --- | --- | --- | --- | --- | --- | --- | --- | --- | --- | --- | --- | --- |
|  |  | 0 | 1 | 2 | 3 | 4 | 5 | 6 | 7 | 8 | 9 | 10 | 11 | 12 | 13 | 14 |
|  |  |  | 0 | 1 | 2 | 3 | 4 | 5 | 6 | 7 | 8 | 9 | 10 | 11 | 12 | 13 |
| Inoculated boar | | | | | | | | | | | | | | | | |
| Boar#191 | Serum | -/-^a^ | NS^b^ | NS | 7.00/+^c^ | NS | NS | -/- | NS | NS | -/- | NS | NS | -/- | NS | -/- |
|  | Oral swab | -/- | 4.75/+ | 5.25/+ | 7.50/+ | 5.25/+ | 5.25/+ | 2.75/+ | -/+ | -/+ | -/+ | -/- | -/- | NS | -/- | -/+ |
|  | Nasal swab | -/- | -/- | -/+ | 6.50/+ | 6.50/+ | 5.50/+ | 4.25/+ | -/+ | -/+ | -/- | -/- | -/- | -/- | -/- | -/- |
|  | VNT | <4 | NS | NS | <4 | NS | NS | 5.6^d^ | NS | NS | 64 | NS | NS | 64 | NS | 45 |
|  | ELISA | - | NS | NS | - | NS | NS | +^e^ | NS | NS | + | NS | NS | + | NS | + |
|  | Clinical score | 0 | 0 | 0 | 0 | 5^f^ | 5 | 5 | 5 | 5 | 5 | 5 | 5 | 5 | 5 | 5 |
| Contact boar and pig | | | | | | | | | | | | | | | | |
| Boar#192 | Serum | NS | -/- | NS | -/- | NS | NS | -/- | NS | NS | -/- | NS | NS | -/- | NS | -/- |
|  | Oral swab | NS | -/- | 4.25/+ | 4.55/+ | 3.30/+ | -/+ | -/+ | -/+ | 3.05/+ | 3.05/+ | -/- | -/- | NS | -/- | 4.25/+ |
|  | Nasal swab | NS | -/- | 2.75/+ | -/- | 3.80/+ | 3.05/+ | -/+ | -/+ | -/- | -/- | -/- | -/- | -/+ | -/- | -/- |
|  | VNT | NS | <4 | NS | <4 | NS | NS | <4 | NS | NS | <4 | NS | NS | <4 | NS | 8 |
|  | ELISA | NS | - | NS | - | NS | NS | - | NS | NS | - | NS | NS | - | NS | + |
|  | Clinical score | NS | 0 | 0 | 0 | 0 | 0 | 0 | 0 | 0 | 0 | 0 | 0 | 0 | 0 | 0 |
| Pig#193 | Serum | NS | -/- | NS | 5.25/+ | NS | NS | -/- | NS | NS | -/- | NS | NS | -/- | NS | -/- |
|  | Oral swab | NS | -/- | 3.25/+ | 5.25/+ | 5.80/+ | 6.00/+ | 5.25/+ | 4.00/+ | 3.00/+ | 2.75/+ | -/+ | -/+ | NS | -/- | -/- |
|  | Nasal swab | NS | -/- | -/- | 3.75/+ | 7.75/+ | 7.80/+ | 5.30/+ | 4.25/+ | 4.00/+ | -/- | -/+ | -/- | -/- | -/- | -/- |
|  | VNT | NS | <4 | NS | <4 | NS | NS | <4 | NS | NS | 16 | NS | NS | 22 | NS | 45 |
|  | ELISA | NS | - | NS | - | NS | NS | + | NS | NS | + | NS | NS | + | NS | + |
|  | Clinical score | NS | 0 | 0 | 0 | 6 | 6 | 6 | 6 | 6 | 6 | 6 | 6 | 6 | 6 | 6 |
| Inoculated pig | | | | | | | | | | | | | | | | |
| Pig#194 | Serum | -/- | NS | NS | 2.25/+ | NS | NS | 3.00/+ | NS | NS | -/- | NS | NS | -/- | NS | -/- |
|  | Oral swab | -/- | -/- | 4.25/+ | 6.25/+ | 5.25/+ | 3.75/+ | 3.75/+ | 3.75/+ | -/+ | -/+ | -/+ | -/+ | -/- | -/+ | -/- |
|  | Nasal swab | -/- | -/- | -/- | -/- | -/+ | -/+ | 7.25/+ | 5.00/+ | 3.00/+ | -/- | -/+ | -/+ | -/- | -/- | -/- |
|  | VNT | <4 | NS | NS | <4 | NS | NS | <4 | NS | NS | 45 | NS | NS | 90 | NS | 64 |
|  | ELISA | - | NS | NS | - | NS | NS | - | NS | NS | + | NS | NS | + | NS | + |
|  | Clinical score | 0 | 0 | 0 | 0 | 0 | 3 | 5 | 6 | 6 | 6 | 6 | 6 | 6 | 6 | 6 |
| Contact pig and boar | | | | | | | | | | | | | | | | |
| Pig#195 | Serum | NS | -/- | NS | -/- | NS | NS | 5.00/+ | NS | NS | -/- | NS | NS | -/- | NS | -/- |
|  | Oral swab | NS | -/- | -/- | -/- | -/- | 3.25/+ | 3.75+ | 5.75+ | 5.75+ | 5.00+ | 3.00+ | -/- | -/- | -/- | -/- |
|  | Nasal swab | NS | -/- | -/- | -/- | -/- | 2.75/- | 3.50/- | 8.00/+ | 7.00/+ | 5.00/+ | -/+ | -/- | -/- | -/- | -/- |
|  | VNT | NS | <4 | NS | <4 | NS | NS | <4 | NS | NS | <4 | NS | NS | 16 | NS | 16 |
|  | ELISA | NS | - | NS | - | NS | NS | - | NS | NS | - | NS | NS | + | NS | + |
|  | Clinical score | NS | 0 | 0 | 0 | 0 | 0 | 0 | 5 | 5 | 6 | 6 | 6 | 6 | 6 | 6 |
| Boar#196 | Serum | NS | -/- | NS | -/- | NS | NS | -/- | NS | NS | -/- | NS | NS | -/- | NS | -/- |
|  | Oral swab | NS | -/- | -/- | -/- | -/- | -/+ | 3.25/+ | 3.75/+ | 3.50/+ | -/+ | -/+ | -/+ | -/+ | -/+ | -/- |
|  | Nasal swab | NS | -/- | -/- | -/- | -/- | -/- | -/- | 2.75/+ | 3.75/- | -/- | -/- | -/- | -/- | -/- | -/- |
|  | VNT | NS | <4 | NS | <4 | NS | NS | <4 | NS | NS | <4 | NS | NS | <4 | NS | <4 |
|  | ELISA | NS | - | NS | - | NS | NS | - | NS | NS | - | NS | NS | - | NS | - |
|  | Clinical score | NS | 0 | 0 | 0 | 0 | 0 | 0 | 0 | 0 | 0 | 0 | 0 | 0 | 1 | 1 |

^a^ Results of virus isolation and RT-PCR assay were shown sequentially. Virus titers were shown as 10^χ^ TCID_50_/mL. "+" means positive results in the RT-PCR assay. "-" means negative results in virus isolation and RT-PCR assay.

^b^ Not sampled.

^c^ Days on which virus isolation and/or RT-PCR were positive are colored orange.

^d^ Days on which VNT was positive are colored yellow.

^e^ Days on which ELISA was positive are colored green.

^f^ Days on which clinical signs were scored are colored blue.
