# Supplementary material for: Risk of transmission of foot-and-mouth disease by wild animals: infection dynamics in Japanese wild boar following direct inoculation or contact exposure
Source: Vet Res. 2022 Oct 22;53:86. doi: 10.1186/s13567-022-01106-0 (PMC9587633; doi:10.1186/s13567-022-01106-0)
Supplement: Supplementary file 5 — Additional file 5. Detection of viral RNA in clinical samples by RT-PCR and of antibodies by VNT and ELISA in animals intradermally inoculated with A/MOG/2013 and in contact animals in Experiment 3. [file 13567_2022_1106_MOESM5_ESM.docx]

**Additional file 5 Detection of viral RNA in clinical samples by RT-PCR and of antibodies by VNT and ELISA in animals intradermally inoculated with A/MOG/2013 and in contact animals in Experiment 3**

| Animal | Clinical sample and assay | Days post-inoculation or days post-contact | | | | | | | | | | | | | | | |
| --- | --- | --- | --- | --- | --- | --- | --- | --- | --- | --- | --- | --- | --- | --- | --- | --- | --- |
|  |  | 0 | 1 | 2 | 3 | 4 | 5 | 6 | 7 | 8 | 9 | 10 | 11 | 12 | 13 | 14 |  |
| Inoculated boar | | | | | | | | | | | | | | | | | |
| Boar#201 | Serum | -/-^a^ | 5.44/+^b^ | 7.25/+ | 6.00/+ | -/- | -/- | -/- | NS^c^ | NS | NS | NS | NS | NS | NS | NS |  |
|  | Oral swab | -/- | 4.05/+ | 7.50/+ | 6.50/+ | 4.75/- | 4.75/- | 5.25/+ | NS | NS | NS | NS | NS | NS | NS | NS |  |
|  | Nasal swab | -/- | -/- | 7.50/+ | 6.00/+ | 2.75/- | 2.75/- | 2.75/+ | NS | NS | NS | NS | NS | NS | NS | NS |  |
|  | VNT | <4 | <4 | <4 | <4 | <4 | <4 | 4^d^ | NS | NS | NS | NS | NS | NS | NS | NS |  |
|  | ELISA | - | - | - | - | - | - | +^e^ | NS | NS | NS | NS | NS | NS | NS | NS |  |
|  | Clinical score | 0 | 1^f^ | 3 | 4 | 5 | 5 | 5 | NS | NS | NS | NS | NS | NS | NS | NS |  |
| Contact pig | | | | | | | | | | | | | | | | | |
| Pig#202 | Serum | -/- | -/- | -/- | -/- | -/- | 4.50/+ | 5.00/+ | 4.00/+ | 1.75/+ | -/- | -/- | -/- | -/- | -/- | -/- |  |
|  | Oral swab | -/- | -/- | 7.55/+ | 4.00/+ | -/- | 2.75/- | 6.00/+ | 5.50/+ | 3.00/+ | 3.05/+ | -/- | -/+ | -/- | -/- | -/- |  |
|  | Nasal swab | -/- | -/- | 6.80/+ | -/+ | -/- | -/- | 4.75/+ | + | + | + | + | + | - | - | - |  |
|  | VNT | <4 | <4 | <4 | <4 | <4 | <4 | <4 | <4 | <4 | 5.6 | 22 | 22 | 32 | 90 | 45 |  |
|  | ELISA | - | - | - | - | - | - | - | - | - | + | + | + | + | + | + |  |
|  | Clinical score | 0 | 0 | 0 | 0 | 0 | 0 | 0 | 2 | 4 | 4 | 4 | 4 | 4 | 4 | 4 |  |
| Pig#203 | Serum | -/- | -/- | -/- | -/- | -/- | -/- | -/- | -/- | -/- | -/- | -/- | -/- | -/- | -/- | -/- |  |
|  | Oral swab | -/- | -/- | 6.30/+ | 6.25/+ | 6.75/+ | 5.00/+ | 5.75/+ | 4.50/+ | 4.50/+ | -/+ | -/- | -/+ | -/- | -/- | -/- |  |
|  | Nasal swab | -/- | -/- | 5.50/+ | 4.75/+ | 2.75/- | -/- | 2.75/- | 3.00/+ | -/+ | -/- | -/+ | -/- | -/- | -/- | -/- |  |
|  | VNT | <4 | <4 | <4 | <4 | <4 | <4 | <4 | <4 | <4 | <4 | <4 | 8 | 16 | 11 | 8 |  |
|  | ELISA | - | - | - | - | - | - | - | - | - | - | - | - | - | - | - |  |
|  | Clinical score | 0 | 0 | 0 | 0 | 0 | 0 | 0 | 0 | 1 | 1 | 1 | 1 | 1 | 1 | 1 |  |
| Inoculated pig | | | | | | | | | | | | | | | | | |
| Pig#204 | Serum | -/- | 5.75/+ | 6.00/+ | 4.75/+ | -/- | -/- | -/- | NS | NS | NS | NS | NS | NS | NS | NS |  |
|  | Oral swab | -/- | 4.75/+ | 6.80/+ | 6.50/+ | 5.50/- | 3.00/+ | 3.00/+ | NS | NS | NS | NS | NS | NS | NS | NS |  |
|  | Nasal swab | -/- | 3.25/+ | 5.25/+ | 5.25/+ | 4.50/- | 4.50+ | -/+ | NS | NS | NS | NS | NS | NS | NS | NS |  |
|  | VNT | <4 | <4 | <4 | <4 | 5.6 | 11 | 11 | NS | NS | NS | NS | NS | NS | NS | NS |  |
|  | ELISA | - | - | - | - | - | - | - | NS | NS | NS | NS | NS | NS | NS | NS |  |
|  | Clinical score | 0 | 1 | 1 | 4 | 4 | 4 | 4 | NS | NS | NS | NS | NS | NS | NS | NS |  |
| Contact boar | | | | | | | | | | | | | | | | | |
| Boar#205 | Serum | -/- | -/- | -/- | -/- | 2.00/- | 2.75/+ | 4.25/+ | 2.75/+ | 1.75/+ | -/+ | -/+ | -/- | -/- | -/- | -/- |  |
|  | Oral swab | -/- | -/- | 4.25/+ | 4.00/+ | -/- | -/- | -/+ | 5.50/+ | 5.00/+ | 4.50/+ | 4.00/+ | 3.75/+ | 4.00/+ | 2.75/+ | -/- |  |
|  | Nasal swab | -/- | -/- | -/- | -/+ | -/- | -/- | 3.00/- | 5.10/+ | 3.35/+ | -/+ | -/+ | -/- | -/- | -/- | -/- |  |
|  | VNT | <4 | <4 | <4 | <4 | <4 | <4 | <4 | <4 | 5.6 | 11 | 45 | 22 | 45 | 22 | 22 |  |
|  | ELISA | - | - | - | - | - | - | - | - | - | - | + | + | + | + | + |  |
|  | Clinical score | 0 | 0 | 0 | 0 | 0 | 0 | 0 | 0 | 0 | 0 | 0 | 0 | 0 | 0 | 0 |  |
| Boar#206 | Serum | -/- | -/- | -/- | 3.75/+ | 3.25/- | 3.75/+ | -/+ | NS | NS | NS | NS | NS | NS | NS | NS |  |
|  | Oral swab | -/- | -/- | 6.25/+ | 3.75/- | 6.25/+ | 5.25/+ | 3.50+ | NS | NS | NS | NS | NS | NS | NS | NS |  |
|  | Nasal swab | -/- | -/- | -/+ | 3.75/- | 6.25/+ | 6.25/+ | 4.00/+ | NS | NS | NS | NS | NS | NS | NS | NS |  |
|  | VNT | <4 | <4 | <4 | <4 | <4 | <4 | <4 | NS | NS | NS | NS | NS | NS | NS | NS |  |
|  | ELISA | - | - | - | - | - | - | - | NS | NS | NS | NS | NS | NS | NS | NS |  |
|  | Clinical score | 0 | 0 | 0 | 0 | 0 | 0 | 0 | NS | NS | NS | NS | NS | NS | NS | NS |  |

^a^ Results of virus isolation and RT-PCR assay were shown sequentially. Virus titers were shown as 10^χ^ TCID_50_/mL. "+" means positive results in the RT-PCR assay. "-" means negative results in virus isolation and RT-PCR assay.

^b^ Not sampled.

^c^ Days on which virus isolation and/or RT-PCR were positive are colored orange.

^d^ Days on which VNT was positive are colored yellow.

^e^ Days on which ELISA was positive are colored green.

^f^ Days on which clinical signs were scored are colored blue.
